# *Bulbophyllum reflexipetalum* (Orchidaceae, Epidendroideae, Malaxideae), a new species from Xizang, China

**DOI:** 10.3897/phytokeys.130.34153

**Published:** 2019-08-29

**Authors:** Ji-Dong Ya, Yong-Jie Guo, Cheng Liu, Jie Cai, Gui-Jun Dong, Hong Jiang, De-Zhu Li

**Affiliations:** 1 Germplasm Bank of Wild Species, Kunming Institute of Botany, Chinese Academy of Sciences, Lanhei Road 132, Heilongtan, Kunming, Yunnan, 650201, China Kunming Institute of Botany, Chinese Academy of Sciences Kunming China; 2 Yunnan Laboratory for Conservation of Rare/Endangered & Endemic Forest Plants, National Forestry and Grassland Administration, Yunnan Academy of Forestry, Lan’an Road 2 Kunming, Yunnan, 650204, China Forestry Bureau of Linzhi Prefecture Linzhi China; 3 The Administration of Nature Reserve, Forestry Bureau of Linzhi Prefecture, Linzhi, 860000, Xizang, China National Forestry and Grassland Administration Kunming China

**Keywords:** Taxonomy, *
Bulbophyllum
*, Section *Umbellata*, Xizang Province, China

## Abstract

*Bulbophyllum reflexipetalum*, a new species from Motuo County, Southeast Xizang, China, is described and illustrated here. This new species belongs to Bulbophyllum sect. Umbellata Bentham & J. D. Hooker, and it is morphologically similar to *B. umbellatum* Lindley, *B. guttulatum* (J. D. Hooker) N. P. Balakrishnan and *B. salweenensis* X.H. Jin, but is distinguished from them by having reflexed petals, base of dorsal sepal with 1 dentate on each side, lip with significantly revolute margin, adaxially with dark brown spots or patches and one longitudinal groove.

## Introduction

*Bulbophyllum* Thouarm (Orchidaceae) is one of the three largest genera in the orchid family, comprising about 2200 species widely distributed in tropical Africa, Asia and America (Lang and Tsi 1987, Pridgeon et al. 2014). There are about 105 species in China (Chen and Vermeulen 2009), with several new species described in recent years (Zhou and Jin 2015, Liu et al. 2016, Zhai et al. 2017). Motuo is an important area in the eastern Himalaya biodiversity hotspot, but the species diversity in this region is poorly known, and some new taxa are discovered and described in recent years (Lai and Jin 2012, Huang et al. 2013, Raskoti et al. 2017). More conservation efforts are needed in this region to counteract the increasing anthropogenic disturbance and destruction. During our field survey in Motuo County, Xizang Autonomous Region, a new Bulbophyllum species of sect. Umbellata Bentham & J. D. Hooker was found in the subtropical broad-leaved forest and described below.

## Materials and methods

Type specimens were collected in Motuo County, Xizang Autonomous Region, China, during a field expedition in 2016. Photographs were taken in field. Shapes, colors and other details given in the description were based on living materials (five individuals). The column and pollinia morphological photographs were taken using an Olympus SZX16. Voucher specimens were deposited at the herbarium of Kunming Institute of Botany, Chinese Academy of Sciences (**KUN**). The conservation status of the new species was evaluated based on the guidelines of the International Union for Conservation of Nature (IUCN 2012).

## Taxonomy

### Bulbophyllum reflexipetalum

Taxon classificationPlantaeAsparagalesOrchidaceae

J.D.Ya, Y.J.Guo & C.Liu sp. nov.

7B2F8158A2985C56B26ACAFA4ECBFDB1

urn:lsid:ipni.org:names:77201398-1

Figure 1 , 2

#### Diagnosis.

*Bulbophyllum reflexipetalum* is similar to *B. salweenensis* X.H. Jin, *B. umbellatum* Lindley and *B. guttulatum* (J. D. Hooker) N. P. Balakrishnan in terms of morphological structure and shape of the flowers. The new species can be distinguished from *B. salweenensis* by the absence of sheaths on the pseudobulbs and rhizome, scape longer than leaf and petals apex mucronate. It can be distinguished from *B. umbellatum* with smaller and flattened void or ovoid-conic pseudobulbs, shorter leaf blade, petals apex caudate. In addition, the new species can be distinguished from *B. guttulatum* with leaf blade oblong and apex emarginated, shorter pedicel and ovary, lip papillae with a single longitudinal ridge.

#### Type.

CHINA. Xizang Autonomous Region: Motuo, subtropical, evergreen broad-leaved forest, 1378 m, 17 Mar 2016, *J.-D. Ya, Y.-J. Guo, Q.-R. Zhang 18HT1419* (holotype: KUN!).

#### Description.

Epiphytic herbs. Rhizome creeping and rooting, 3–4 mm in diam. Roots arising from the nodes, 0.5–1.5 mm in diam. Pseudobulbs often 1–2 cm apart on rhizome, flattened ovoid or ovoid-conic, ca. 10–16 × 8–15 mm, with a terminal leaf. Leaf blade oblong, 50–65 × 15–20 mm, leathery, apex obtuse and emarginate, base narrowing into a petiole; petiole 10–20 mm. Scape arising from the base of pseudobulb, 13–20 cm long, longer than leaf; umbellate, often 3–4-flowered; peduncle ca. 1.5 mm in diam, with 3 tubular sheaths; floral bract lanceolate, acuminate, concave, ca. 6.3 × 2.0 mm; peduncle and ovary ca. 10–15 mm long. Flowers greenish yellow, sepals and petals with dark brown spots; lip greenish yellow, adaxially with dark brown spots or patches. Dorsal sepal ovate, concave, apex obtuse, base with 1 dentate on each side, ca. 8.10 × 4.95 mm, 5-veined; lateral sepals falcate-lanceolate, base adnate to column foot, twisted inward near base, ca. 10.30 × 5.26 mm, 5-veined; Petals reflexed, broadly ovate-triangular, ca. 3.23 × 2.52 mm, 1-veined, margin entire, apex mucronate; lip deflexed, triangular-lingulate, ca. 4.02 × 2.94 mm, base subcordate with papillae on the edge, margin revolute, apex obtuse, adaxially with a longitudinal ridge from base to apex, abaxially with a longitudinal groove; column ca. 3.25 mm, with deltoid, rounded wings along lower margins, stelidia deltoid, ca. 1 mm, foot ca. 6.86 mm, apex attached to lip; anther cap subglobose with many longitudinal lamellae when dry. pollinia 4, in 2 pairs, 0.72–0.93 mm, yellow, ovate, waxy, attached to sticky substance. Fl. February–March.

#### Etymology.

The specific epithet “*reflexipetalum*” refers to reflexed petals of this new species.

#### Vernacular name.

Fan Ban Juan Ban Lan (Chinese pronunciation); 反瓣卷瓣兰 (Chinese name).

#### Distribution and habitat.

*Bulbophyllum reflexipetalum* is currently known only from the type locality of Motuo, Southeast Xizang, China. It is a predominantly epiphytic species that grows on tree trunks, under broadleaf evergreen forest at the elevation between 1300 m and 1400 m.

#### Conservation status.

During our 2-weeks field survey, only 2 populations were found. As this new species may also grow in the broadleaf evergreen forest of vicinity region, we regard its status as Data Deficient (IUCN 2012).

**Figure 1.**
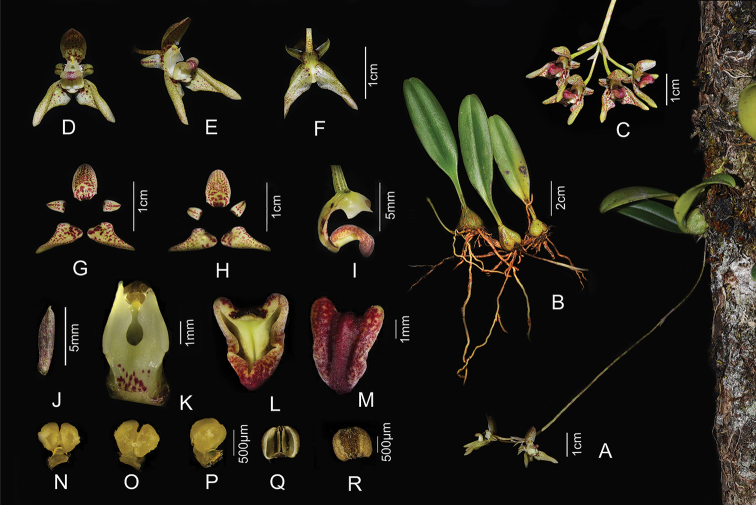
*Bulbophyllum reflexipetalum* sp. nov. **A** habitat **B** plant **C** inflorescence **D** front view of flower **E** lateral view of flower **F** dorsal view of flower **G** adaxial sepals and petals **H** abaxial sepals and petals **I** lateral view of column and lip **J** bract **K** front view of column **L** dorsal view of labellum **M** front view of labellum **N** dorsal view of pollinarium **O** front view of pollinarium **P** lateral view of pollinarium **Q** abaxial anther cap **R** adaxial anther cap (Photographed by J.-D. Ya).

**Figure 2.**
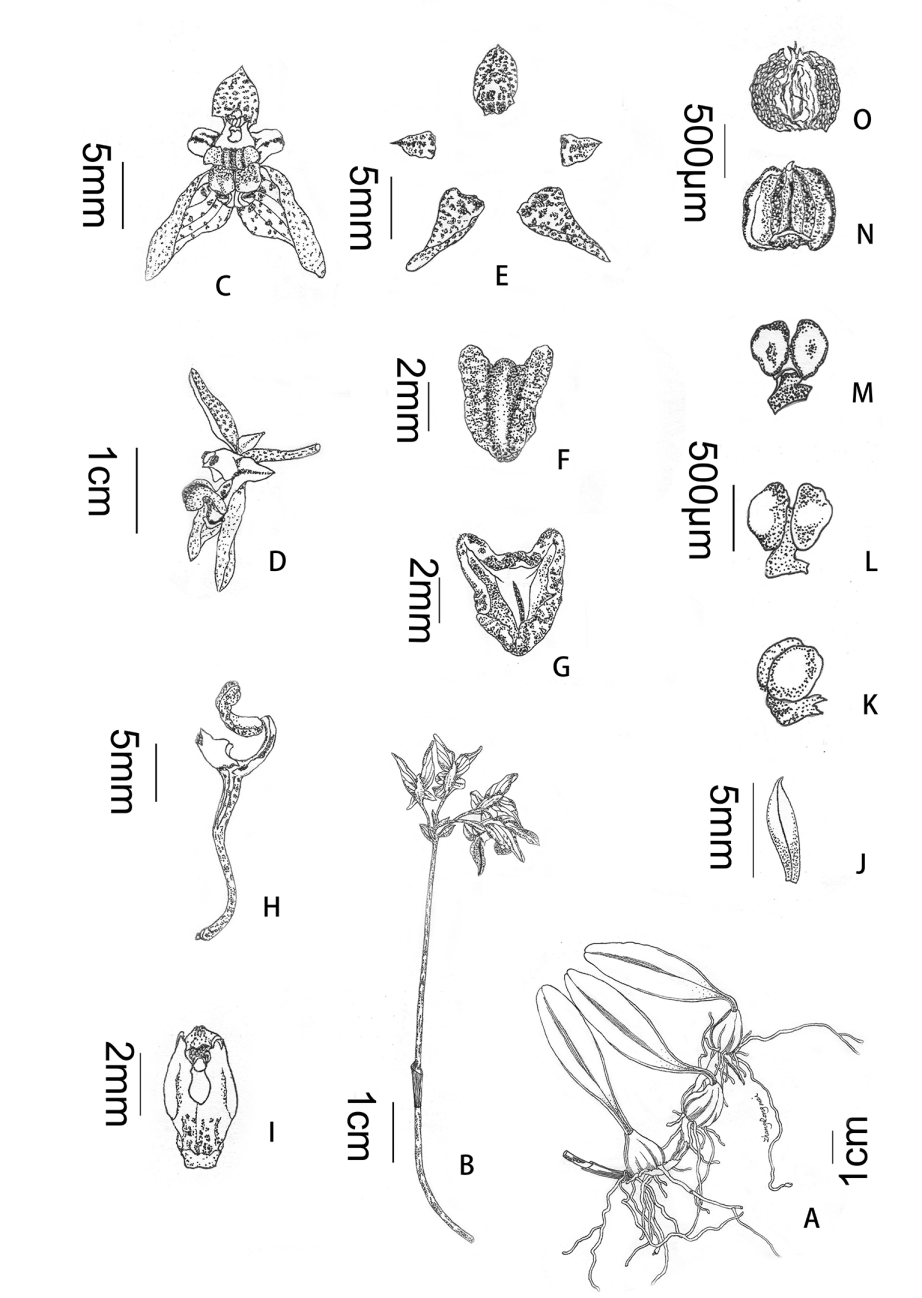
*Bulbophyllum reflexipetalum* sp. nov. **A** plant **B** inflorescence **C** front view of flower **D** lateral view of flower **E** adaxial sepals and petals **F** front view of labellum **G** dorsal view of labellum **H** lateral view of column and lip **I** front view of column **J** bract **K** lateral view of pollinarium **L** front view of pollinarium **M** dorsal view of pollinarium **N** abaxial anther cap **O** adaxial anther cap (Drawn by Rong-Mei Zhang).

## Discussion

**Figure 3.**
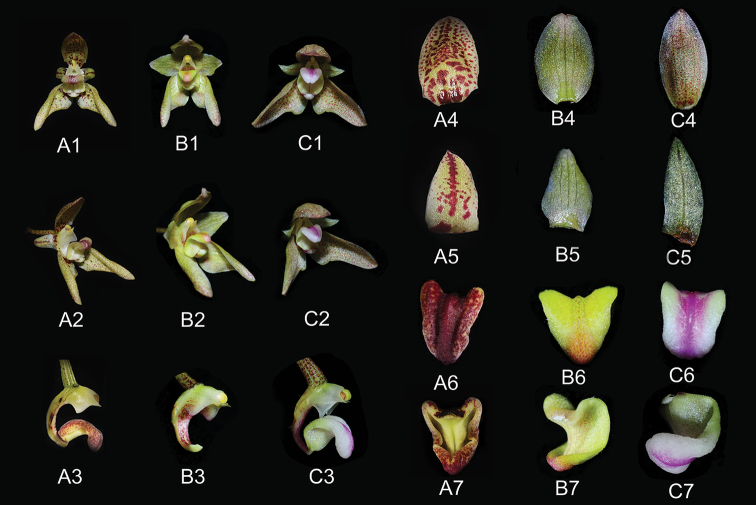
**A***Bulbophyllum reflexipetalum***B***B. umbellatum***C***B. salweenensis***1** front view of flower **2** lateral view of flower **3** lateral view of column and lip **4** dorsal sepal **5** petal **6** front view of column **7** dorsal or lateral view of labellum (Photographed by J.-D. Ya & H. Jiang).

*Bulbophyllum reflexipetalum* belongs to sect. Umbellata based on the umbellate inflorescence (Chen and Vermeulen 2009). Morphologically, this new species is similar to *B. salweenensis*, *B. umbellatum* and *B. guttulatum* in terms of vegetative morphology and shape of the flowers (Figure 3), but is easily recognized by its reflexed petals, the dentate of dorsal sepal and the revolute margin of lip (Table 1). Beyond that, *B. reflexipetalum* can be distinguished from *B. salweenensis* by the absence of sheaths on the pseudobulbs and rhizome (vs. pseudobulbs and rhizome with sheaths), scape longer than leaf and petals apex mucronate (vs. the scape shorter than leaf); it differs from *B. umbellatum* with smaller and flattened void or ovoid-conic pseudobulbs, shorter leaf blade, petals apex caudate (vs. obtuse-rounded petals) ; and it differs from *B. guttulatum* with leaf blade oblong and apex emarginated (vs. leaf blade elliptic-oblong and apex rounded), shorter pedicel and ovary, lip papillae with a single longitudinal ridge (lip with three ridges in *B. guttulatum*) (Chen and Vermeulen 2009, Zhou and Jin 2015).

**Table 1.** Morphological comparison of *Bulbophyllum reflexipetalum* and its closely related species.

Characters	*Bulbophyllum reflexipetalum*	*B. salweenensis*	*B. umbellatum*	*B. guttulatum*
Pseudobulbs	flattened ovoid or ovoid-conic, without sheaths	ovoid-conic, with sheaths	ovoid-conic or narrowly ovoid, without sheaths	ovoid or ovoid-conic, without sheaths
	10–16 × 8–15 mm	10–40 × 4–6 mm	20–25 × 7–10 mm	13–35 × 10–20 mm
Leaf blade	oblong, apex emarginate, 5–6.5 × 1.5–2 cm	narrowly oblong, apex emarginate, 4–16 × 0.5–2.5 cm	oblong, apex emarginate, 8–19× 1.3–2.8 cm	elliptic-oblong, apex rounded, 7–14 × ca. 3 cm.
Scape	13–20 cm, longer than leaf	4–9 cm, shorter than leaf	10–15 cm	8–12 cm
Pedicel and ovary	10–15 mm	ca. 10 mm	ca. 20 mm	ca. 25 mm
Flowers	greenish yellow with dark brown spots	greenish yellow with purplish red spots	dark greenish yellow or brown with purplish apex	yellow with red spots
Dorsal sepal	Ovate, apex obtuse, base with 1 dentate	ovate, acuminate, entire.	ovate, concave, apex acute	broadly ovate, apex mucronulate
Petals	Reflexed, broadly ovate-triangular, ca.3.23 × 2.52 mm, apex mucronate	lanceolate, denticulate, ca. 5 × 2 mm, apex caudate	ovate, ca. 7 × 5 mm, obtuse-rounded	broadly ovate-triangular, ca. 4.5 × 4 mm, apex mucronate
Lip	greenish yellow, adaxially with dark brown spots or patches	whitish purple at base, pink at apex, keel pink.	white	white with purple spots
	triangular-lingulate, apex obtuse, ca. 4.02×2.94 mm	lingulate, apex obtuse,	lingulate, apex obtuse,	subovate, apex emarginated, 5 × 3.4 mm
		ca. 8×3 mm;	ca. 8×3 mm;	
	adaxially papillae with a longitudinal ridge, margin revolute, abaxial with a longitudinal groove;	adaxially papillae with a longitudinal ridge	adaxially papillae with a longitudinal ridge	adaxially with 3 longitudinal ridges

## Supplementary Material

XML Treatment for Bulbophyllum reflexipetalum
